# The Immediate and Chronic Influence of Spatio-Temporal Metaphors on the Mental Representations of Time in English, Mandarin, and Mandarin-English Speakers

**DOI:** 10.3389/fpsyg.2013.00142

**Published:** 2013-04-09

**Authors:** Vicky Tzuyin Lai, Lera Boroditsky

**Affiliations:** ^1^Neurobiology of Language Department, Max Planck Institute for PsycholinguisticsNijmegen, Netherlands; ^2^Department of Psychology, Stanford UniversityStanford, CA, USA

**Keywords:** time, space, metaphor, Mandarin, bilingualism

## Abstract

In this paper we examine whether experience with spatial metaphors for time has an influence on people’s representation of time. In particular we ask whether spatio-temporal metaphors can have both chronic and immediate effects on temporal thinking. In Study 1, we examine the prevalence of ego-moving representations for time in Mandarin speakers, English speakers, and Mandarin-English (ME) bilinguals. As predicted by observations in linguistic analyses, we find that Mandarin speakers are less likely to take an ego-moving perspective than are English speakers. Further, we find that ME bilinguals tested in English are less likely to take an ego-moving perspective than are English monolinguals (an effect of L1 on meaning-making in L2), and also that ME bilinguals tested in Mandarin are more likely to take an ego-moving perspective than are Mandarin monolinguals (an effect of L2 on meaning-making in L1). These findings demonstrate that habits of metaphor use in one language can influence temporal reasoning in another language, suggesting the metaphors can have a chronic effect on patterns in thought. In Study 2 we test Mandarin speakers using either horizontal or vertical metaphors in the immediate context of the task. We find that Mandarin speakers are more likely to construct front-back representations of time when understanding front-back metaphors, and more likely to construct up-down representations of time when understanding up-down metaphors. These findings demonstrate that spatio-temporal metaphors can also have an immediate influence on temporal reasoning. Taken together, these findings demonstrate that the metaphors we use to talk about time have both immediate and long-term consequences for how we conceptualize and reason about this fundamental domain of experience.

## Introduction

To represent time, many cultures around the world rely on space. People spatialize time in cultural artifacts like graphs, time-lines, orthography, clocks, sundials, hourglasses, and calendars. We gesture temporal relations, and rely heavily on spatial words (e.g., *forward*, *back*, *long*, *short*) to talk about the order and duration of events (e.g., Clark, 1973; Traugott, 1978; Lakoff and Johnson, 1980). People’s private mental representations of time also appear to be based in space: irrelevant spatial information readily affects people’s judgments of temporal order and duration (Boroditsky, 2000; Boroditsky and Ramscar, 2002; Matlock et al., 2005; Núñez et al., 2006; Casasanto and Boroditsky, 2008; Boroditsky and Gaby, 2010), and people seem to implicitly and automatically generate spatial representations when thinking about time (Gevers et al., 2003; Torralbo et al., 2006; Santiago et al., 2007; Ishihara et al., 2008; Weger and Pratt, 2008; Fuhrman and Boroditsky, 2010; Miles et al., 2010).

However, the particular ways that time is spatialized differ across languages and cultures. Research done around the world has uncovered dramatic variability in representations of time across cultures and groups. Several aspects of linguistic, cultural, and personal experience appear to shape people’s temporal reasoning, such as: (1) the pattern of spatial metaphors that people use to talk about time (Boroditsky, 2001; Casasanto et al., 2004; Núñez and Sweetser, 2006; Boroditsky et al., 2011; Fuhrman et al., 2011), (2) the set of spatial representations and reference frames that are available for co-opting for thinking about time (either in the linguistic or cultural environment more generally, or in the immediate context more specifically) (Boroditsky, 2000; Boroditsky and Ramscar, 2002; Matlock et al., 2005; Núñez et al., 2006; Boroditsky and Gaby, 2010), (3) organizational patterns in cultural artifacts (e.g., writing direction) (Tversky et al., 1991; Fuhrman and Boroditsky, 2010; Ouellet et al., 2010; Bergen and Lau, 2012), and (4) aspects of cultural or individual disposition, age, and experience (Gonzalez and Zimbardo, 1985; Carstensen, 2006; Ji et al., 2009).

In this paper we focus on the role that spatial metaphors play in constructing representations of time across languages, with a particular focus on English and Mandarin. When talking about time in English, we can look *forward* to the challenges *ahead* of us, move meetings *back*, or fall *behind* on deadlines. In Mandarin one can fondly remember dinner from the *front day* (the day before yesterday) or eagerly anticipate the *down month* (next month). Depending on the language we’re speaking we might talk about the future as if it lies ahead of us (in English) or below us (in Mandarin Chinese). Do such differences in metaphorical language influence how people mentally organize the domain of time? If so, is such influence momentary, long lasting, or both? We investigate these questions by comparing spatial representations for time in people who can speak Mandarin, English, or Mandarin and English, in two studies.

In Study 1, we test whether habits of metaphor use in one language can influence temporal reasoning in another language. Such a finding would suggest that patterns in metaphor use can have chronic effects on patterns in thought. We measure the relative cognitive salience of ego-moving and time-moving conceptualizations for English and Mandarin speakers, and examine whether and how Mandarin-English (ME) bilinguals integrate the patterns from their two languages into their temporal thinking.

In Study 2 we examine whether using different metaphors within a language invites different representations of time in-the-moment. Specifically, we ask whether Mandarin speakers flexibly re-organize time along the front-back or up-down axis depending on whether they are processing front-back or up-down metaphors for time.

## Study 1: Chronic Effects of Metaphor Use

### Background

In English, two dominant spatial metaphors are used to sequence events in time (McTaggart, 1908; Clark, 1973; Lakoff and Johnson, 1980). The first is the ego-moving metaphor, in which time is conceived as a stationary path and the “ego” moves along the timeline toward the future as in (1a). The second is the time-moving metaphor, in which the observer is stationary and time is conceived moving past the observer from the future to the past as in (1b).

**Table:** 

(1)	a. We are approaching the deadline.
	b. The deadline is approaching.

Time-moving and ego-moving metaphors are also available in Mandarin (Table 1). Some researchers have suggested that time-moving metaphors in Mandarin are more frequent and less restricted than ego-moving metaphors, making time-moving conceptualizations the dominant representations of time (Huang, 1978; Tai, 1993; Alverson, 1994; Yu, 1998; Ahrens and Huang, 2002; Dong, 2004; but see Gong, 2009; Zhou, 2001).

**Table 1:** **Examples of spatio-temporal metaphors in Mandarin**.

(1)	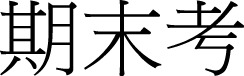	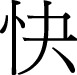	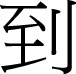	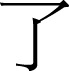		(2)	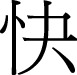	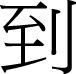	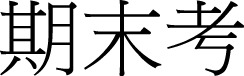	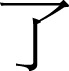	
	qi-mo-kao	kuai	dao	le			kuai	dao	qi-mo-kao	le	
	final-exam	fast	arrive	particle-le			fast	arrive	final-exam	particle-le	
	“The finals are fast approaching.”						“(Pro-drop we) are fast approaching the finals.”				

(3)	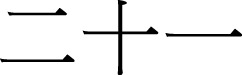	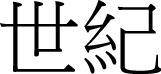	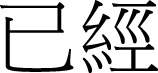	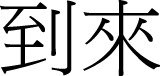		(4)	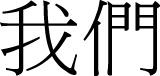	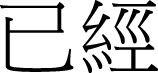	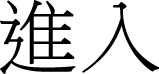	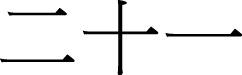	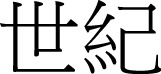
	er-shi-yi	shi-ji	yi-jing	dao-lai			wo-men	yi-jing	jin-ru	er-shi-yi	shi-ji
	twenty-one	century	already	come			we	already	enter	twenty-one century	
	“The 21st century has come.”						“We have entered the 21st century”				

(5)	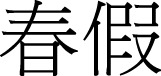	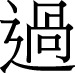	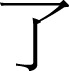			(6)	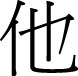 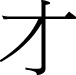	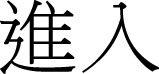	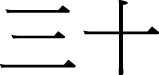		
	chun-jia	guo	le				ta cai	jing-ru	san-shi		
	spring-vacation	pass	aspectual-le				he just	enter	three-ten		
	“The spring break has passed.”						“He just entered the thirties.”				

(7a)	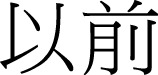				(7b) 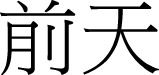				(7c) 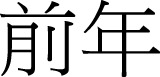		
	yi-qian				qian-tian				qian-nian		
	to-front				front day				front-year		
	“before”				“the day before yesterday”				“the year before last year”			

(8a)	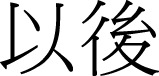				(8b) 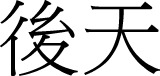				(8c) 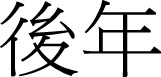		
	yi-hou				hou-tian				hou-nian		
	to-back				back day				back year		
	“after”				“the day after tomorrow”				“the year after the next year”		

(9)	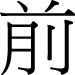	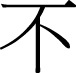	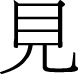		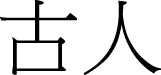	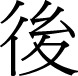		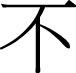	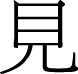	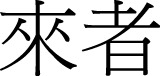	
	qian	bu	jian		gu-ren,	hou		bu	jian	lai-zhe	
	front	no	see		ancient-person	back		no	see	come-person	
	“(Pronoun-drop I) can’t see any predecessor before me, or any new comer behind me”										

(10a)	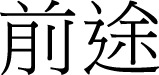			(10b) 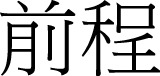				(10c) 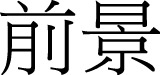			
	qian-tu			qian-cheng				qian-jing			
	front-path			front-journey				front-view			
	“future”			“future”				“outlook”			

(11a)	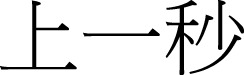	(11b) 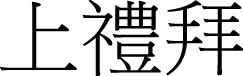			(11c) 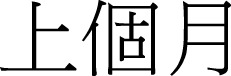			(11d) 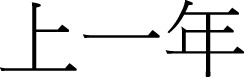			(11e) 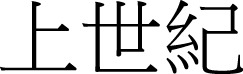
	shang yi miao	shang li-bai			shang ge yue			shang yi nian			shang shi-ji
	up one second	up week			up classifier-ge month			up one year			up century
	“last second”	“last week”			“last month”			“last month”			“last century”

(12a)	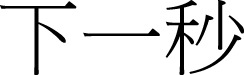	(12b) 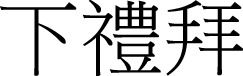			(12c) 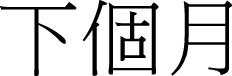			(12d) 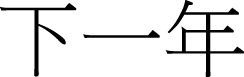			(12e) 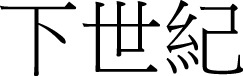
	xia yi miao	xia li-bai			xia ge yue			xia yi nian			xia shi-ji
	down one second	down week			down classifier-ge month			down one year			down century
	“next second”	“next week”			“next month”			“next month”			“next century”

The first goal of our paper is to test empirically whether Mandarin speakers are less likely to assume the ego-moving perspective on time than are English speakers, and whether and how bilinguals exposed to both languages may assimilate the patterns of both languages into their temporal thinking.

We tested Mandarin and English monolinguals and ME bilinguals (some tested in English, and some in Mandarin) on the same questions. Testing bilinguals allows us to ask two questions: (1) whether knowing Mandarin affects how ME bilinguals understand spatio-temporal metaphors in English, and (2) whether learning English affects how ME bilinguals understand spatio-temporal metaphors in Mandarin. That is, does L1 have an effect on how people conduct meaning-making in L2, and vice versa can L2 have an effect on how people conduct meaning-making in L1?

### Participants

Participants gave informed consent and were tested on one of two questions about time. One set of participants was tested on a question about rescheduling a meeting. The other set was tested on a question about resetting a clock. After the participants completed the study, they reported their language proficiency by filling out a language background questionnaire, listing the languages they speak, and indicating how proficient they are in each (on a scale of 1 to 5; with a score of 0 assigned to languages that participants reported not speaking at all). A number of our participants reported fluency in Cantonese as well as Mandarin. In order to focus our studies on Mandarin, we excluded all participants with a fluency in Cantonese greater than 0.

#### The meeting question

One hundred and seventy two people were included in this part of the study, including 66 native English speakers residing in the US (English proficiency = 5, Mandarin proficiency = 0, mean age = 19.9), 51 native Mandarin speakers residing in Taiwan (English proficiency = 1.0, Mandarin proficiency = 5.0, mean age = 22.5), and 55 ME bilinguals residing in the US (English proficiency = 4.02, Mandarin proficiency = 4.95, mean age = 24.0).

#### The clock question

Ninety-one people participated in this part of the study, including 28 native English speakers residing in the US (English proficiency = 5, Mandarin proficiency = 0, mean age = 27.3), 24 native Mandarin speakers residing in Taiwan (English proficiency = 1.71, Mandarin proficiency = 5.00, mean age = 20.1), and 39 ME bilinguals residing in the US (English proficiency = 4.24, Mandarin proficiency = 4.81, mean age = 25.9).

### Materials and methods

#### The meeting question

The question administered to this group is about moving a meeting (Table 2). This question is ambiguous with two possible correct answers: Monday or Friday. If one takes an ego-moving perspective, then forward is in the direction of motion of the observer, hence the meeting should move from Wednesday to Friday. If one takes the time-moving perspective, then forward is in the direction of motion of time, hence the meeting should move from Wednesday to Monday. This question has been used in many previous studies to assess whether individuals take an ego-moving or time-moving perspective on time (McGlone and Harding, 1998; Boroditsky, 2000; Boroditsky and Ramscar, 2002).

**Table 2:** **The meeting question in English (top) and Mandarin (bottom)**.

Next Wednesday’s meeting has been moved forward two days. What day is the meeting now that it has been rescheduled?							
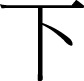	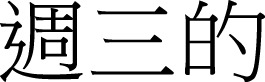	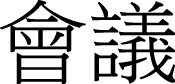	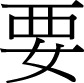	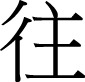	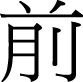	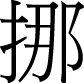	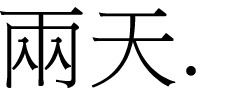
Xia	zhou-san-de	hui-yi	yao	wang	qian	nuo	liang-tian.
down	Wednesday’s	meeting	will	toward	front	move	two days.
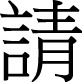	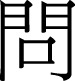	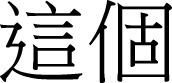	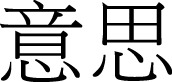	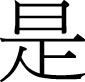	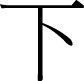	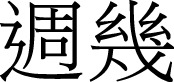	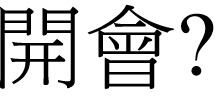
qing	wen	zhe-ge	yi-si	shi	xia	zhou-ji	kai-hui?
Please	ask	this	meaning	is	down	week which	meet?

The native Mandarin-speaking group was tested in Mandarin. The native English and the ME bilingual groups were tested in English. This allows us to test for the effect of L1 on meaning-making in L2, by comparing English monolinguals and ME bilinguals on the same task, tested using the very same materials in English.

#### The clock question

The question administered to this group is about changing the time on a clock (Table 3). Possible correct answers would be 12:00 p.m. (time-moving perspective) or 2:00 p.m. (ego-moving perspective). The native English group was tested in English. The native Mandarin and the ME bilinguals groups were tested in Mandarin. This comparison allows us to test the effect of L2 on meaning-making in L1, by comparing Mandarin monolinguals and ME bilinguals on the same task, tested using the very same materials in Mandarin.

**Table 3:** **The “clock” question in English (top) and Mandarin (bottom)**.

Suppose the clock says it is 1pm now. You need to move it one hour forward. What time will it be adjusted to?									
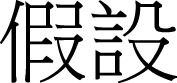	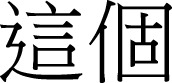	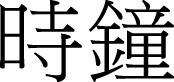	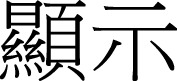	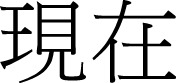	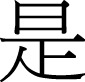	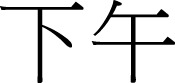	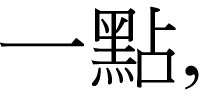		
jia-she	zhe-ge	shi-zhong	xian-shi	xian-zai	shi	xia-wu	yi-dian,		
suppose	this	clock	show	now	is	afternoon	one,		
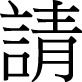	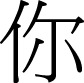	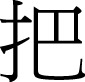	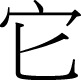	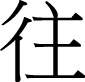	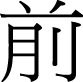	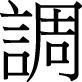	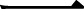	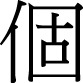	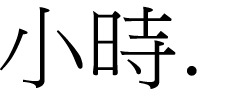
Qing	ni	ba	ta	wang	qian	tiao	yi	ge	xiao-shi
please	you	make	it	toward	forward	adjust	one	classifier-ge	hour.
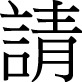	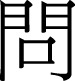	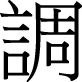	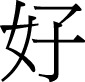	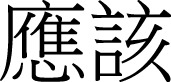	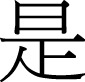	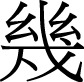	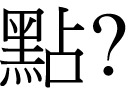		
qing	wen	tiao	hao	ying-gai	shi	ji	dian?		
Please	ask	adjust	ready	should	is	which	hour?		

### Results

**Figure 1:**
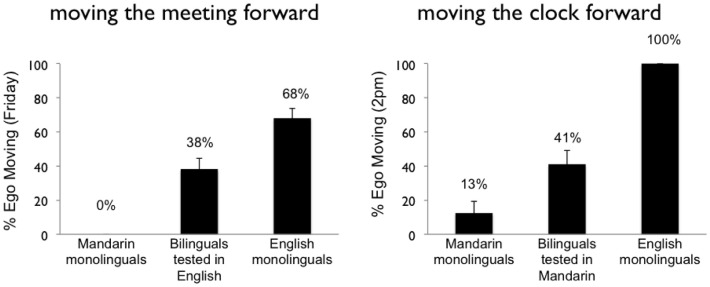
**Results of Study 1**. The *y*-axis indicates the percentage of participants who answered that the meeting has been moved to Friday (left panel) or that the clock should be reset to 2PM (right panel), indicating an ego-moving perspective.

Results are summarized in Figure 1. In brief, we find that English speakers are indeed more likely to take an ego-moving perspective than are Mandarin speakers. Further we find both effects of L1 on L2, and interestingly, also the other way around, L2 on L1.

#### Effects of L1 on L2: The meeting question

When asked the question about next Wednesday’s meeting, English monolinguals were more likely to take the ego-moving perspective and say that the meeting moved to Friday than were ME bilinguals, who were in turn more likely to say Friday than were Mandarin monolinguals (68.2, 38.2, and 0% said Friday respectively). Each group’s pattern of responses differed significantly from the others (English monolinguals vs. ME bilinguals, χ^2^ = (1, *N* = 121) = 9.7, *p* < 0.005; English monolinguals vs. Mandarin monolinguals, χ^2^ = (1, *N* = 117) = 53.7, *p* < 0.0001, Yates-corrected; ME bilinguals vs. Mandarin monolinguals, χ^2^ = (1, *N* = 106) = 22.0, *p* < 0.0001, Yates-corrected). Of course, the difference between the participants tested in English and those tested in Mandarin could simply be due to unavoidable differences in the linguistic format of the question between the two languages. The more telling comparison is that between the English monolinguals and the ME bilinguals, both of whom were tested on the same linguistic stimuli in English. The finding that ME bilinguals interpreted the question about Wednesday’s meeting differently from the native English speakers (and in a direction consistent with the results for the Mandarin monolinguals tested in Mandarin) suggests that they were importing conceptual structures more common in L1 into their understanding of metaphors in L2.

We further interrogated the data from the English monolinguals and the ME bilinguals in a logistic regression, with Mandarin Proficiency as a predictor variable. We found that Mandarin proficiency predicted participants’ time interpretation, β = −0.250, Wald = 10.427, *p* < 0.001. Participants who were more proficient in Mandarin were less likely to take an ego-moving perspective on time.

#### Effects of L2 on L1: The clock question

When asked the question about resetting the clock, English monolinguals were again more likely to take an ego-moving perspective (and say that the clock should be reset to 2:00 p.m.) than were ME bilinguals, who were in turn more likely to do so than were Mandarin monolinguals (100.0, 41.0, and 12.5% resetting to 2:00 p.m. respectively). Each group’s pattern of responses differed significantly from the others [English monolinguals vs. ME bilinguals, χ^2^ = (1, *N* = 67) = 22.6, *p* < 0.0001, Yates-corrected; English monolinguals vs. Mandarin monolinguals, χ^2^ = (1, *N* = 52) = 37.5, *p* < 0.0001, Yates-corrected; ME bilinguals vs. Mandarin monolinguals, χ^2^ = (1, *N* = 63) = 4.465, *p* < 0.05, Yates-corrected]. Of course, the difference between the participants tested in English and those tested in Mandarin could arise simply due to unavoidable differences between the linguistic forms of the question in the two languages. The more telling comparison is that between the Mandarin monolinguals and the ME bilinguals, both of whom were tested on the same stimuli in Mandarin. The finding that ME bilinguals interpreted the question about the clock differently from the monolingual Mandarin speakers (and in a direction more consistent with the results for the English monolinguals tested in English) suggests that they were importing common conceptual structures from their linguistic/cultural experience in L2 into L1.

We further interrogated the data from the Mandarin monolinguals and the ME bilinguals in a logistic regression, with English Proficiency as a predictor variable. We found that English proficiency predicted participants’ time interpretation, β = 0.609, Wald = 6.982, *p* < 0.01). Participants who were more proficient in English were more likely to take an ego-moving perspective on time.

One potential concern is that ME bilinguals included in this study differed from the Mandarin monolinguals not only in that the bilinguals had higher proficiency in English, but also in the Test location. The bilinguals were tested in the US whereas the Mandarin monolinguals were tested in Taiwan. Indeed, in a logistic regression conducted on data from Mandarin monolinguals and ME bilinguals, Test location was a significant predictor of people’s time perspective, β = 1.583, Wald = 5.146, *p* < 0.05. Likewise, in bivariate correlations, both English proficiency and Test location were predictive of people’s time perspective [English Proficiency: *r*(63) = 0.353, *p* < 0.01; Test location: *r*(63) = 0.294, *p* < 0.05]. (Mandarin proficiency was not a significant predictor in these analyses).

To be able to separate out the influence of English proficiency from that of Test location, we further interrogated the data from the ME bilinguals and Mandarin monolinguals in a set of partial correlation analyses. These analyses were designed to examine whether the testing location (Taiwan vs. US) rather than English proficiency may have been the driving force behind the differences between the two groups of Mandarin speakers in answering the clock question. When Test location and Mandarin proficiency were controlled for, English proficiency still predicted participants’ answers to the clock question, *r*(59) = 0.219, *p* < 0.05 (one-tailed: as predicted higher English proficiency was correlated with more ego-moving responses). When language proficiency (English and Mandarin) was controlled for, Test location did not independently predict participants’ answers to the clock question, *r*(59) = 0.017, *p* = 0.449. These results suggest that native Mandarin speakers’ proficiency in English (and prior experience with and familiarity with English time metaphors) affects how likely they are to construct ego-moving representations of time (even when tested entirely in Mandarin). That is, there is an effect of L2 experience on meaning-making in L1.

### Discussion

In this study we tested the relative cognitive salience of ego-moving and time-moving conceptualizations for English and Mandarin speakers. We asked English and Mandarin speakers what it would mean to move a meeting *forward* and set a clock *forward*. In both cases Mandarin speakers interpreted the temporal *forward* as change to an earlier time (Monday, 12:00 p.m.), a pattern consistent with the time-moving perspective. English speakers were more likely than Mandarin speakers to interpret the temporal *forward* as change to a later time (Friday, 2:00 p.m.), a pattern consistent with the ego-moving perspective. These results are consistent with the hypothesis that Mandarin speakers are more likely to take a time-moving perspective on time than are English speakers.

Of course, because the two groups were tested on questions formulated in different languages, it is difficult to know how much of the difference was driven by more general patterns in conceptualization of time in the two groups, and how much might be attributable to unavoidable differences in how the specific questions were formulated in the two languages.

To overcome this difficulty we tested ME bilinguals in English and compared their results to those of English monolinguals. Testing English monolinguals and ME bilinguals on exactly the same question formulated in English allowed us to test whether prior experience speaking Mandarin pre-disposes the ME bilinguals to interpret the English formulation in a more time-moving fashion than do English monolinguals. Indeed, we find that ME bilinguals are less likely to take an ego-moving perspective when understanding English temporal metaphors than are English monolinguals, even when both groups are tested on the identical question in English. This finding reveals how patterns in one’s native language can shade the construction of meaning in a second language.

Taking another approach to this question, we tested ME bilinguals in Mandarin and compared their results to those of Mandarin monolinguals. Testing Mandarin monolinguals and ME bilinguals on exactly the same question formulated in Mandarin allowed us to test whether experience speaking English pre-disposes the ME bilinguals to interpret the Mandarin formulation in a more ego-moving fashion than do Mandarin monolinguals. Indeed, we find that ME bilinguals are more likely to take an ego-moving perspective when understanding Mandarin temporal metaphors than are Mandarin monolinguals, even when both groups are tested on the identical question in Mandarin. This finding reveals how patterns in one’s second language can shade the construction of meaning in one’s native language.

It appears that for bilinguals, both languages hold sway on thinking. That is, there are influences of the first language on conceptualizing time in the second language, and of the second language on conceptualizing time in the first language (see also Brown and Gullberg, 2008, 2010; Lai et al., in press).

In future studies, it would be interesting to compare data from ME bilinguals tested either in English or in Mandarin on the same question, and to compare these results to the two groups of monolinguals. These comparisons would allow us to measure both the contribution of having learned another language (in terms of how much bilinguals deviate from monolinguals of either language) and the contribution of the current linguistic context (in terms of how much bilinguals’ responses differ when tested in Mandarin as opposed to English).

## Study 2: Immediate Effects of Metaphor Use

### Background

In addition to using horizontal terms to talk about time, Mandarin speakers also frequently use vertical terms like *shang* “up” and *xia* “down” to talk about the order of temporal events (2a–d) (Huang, 1978; Scott, 1989; Alverson, 1994; Chun, 1997a,b; Yu, 1998; Liu and Zhang, 2009).

**Table:** 

(2)	a.	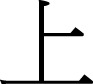	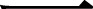	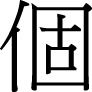	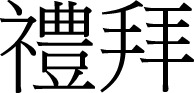
		shang	yi	ge	li-bai
		up	one	classifier-ge	week
		“Last week”			
	b.	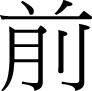	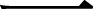	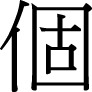	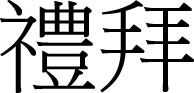
		qian	yi	ge	li-bai
		front	one	classifier-ge	week
		“Last week”			
	c.	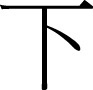	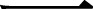	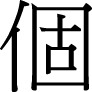	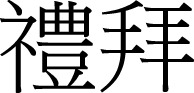
		xia	yi	ge	li-bai
		down	one	classifier-ge	week
		“Next week”			
	d.	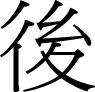	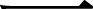	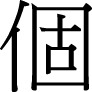	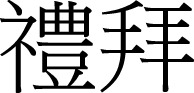
		hou	yi	ge	li-bai
		back	one	classifier-ge	week
		“Next week”			

Previous work has examined whether differences in the background frequency of up-down time metaphors between English and Mandarin predict how English and Mandarin speakers tend to spatialize time. The findings across a variety of linguistic and non-linguistic paradigms suggest that Mandarin speakers are more likely to spatialize time vertically than are English speakers (Boroditsky et al., 2011; Fuhrman et al., 2011; Miles et al., 2011; Bergen and Lau, 2012). However, attributing this cross-linguistic difference in spatialization to differences in metaphor is somewhat complicated because of the concomitant differences in writing direction, which may be responsible for at least some of the cross-cultural differences in spatializing time (e.g., see Bergen and Lau, 2012). One approach to overcome this difficulty is to directly manipulate metaphors within a language to examine whether metaphors can in-the-moment influence how people spatialize time. The fact that Mandarin uses both front-back and up-down metaphors frequently allows us an opportunity to ask this question.

In this section we examine whether metaphor use plays a causal in-the-moment role in how people construct representations of time. Specifically, we ask whether Mandarin speakers flexibly re-organize time along the front-back or up-down axis depending on whether they are processing front-back or up-down metaphors for time. This allows us to test whether Mandarin speakers are sensitive to the spatial meaning in up-down and front-back temporal metaphors as they process them in natural language. If the spatio-temporal metaphors are psychologically dead and no longer carry a spatial meaning, then one might not expect any consequences for how people spatialize time in-the-moment. However, if processing these highly conventionalized spatio-temporal metaphors evokes spatial meaning in people’s minds, then we may see a difference in how Mandarin speakers spatialize time when processing front-back vs. up-down metaphors.

### Participants

Ninety-eight ME bilinguals participated in the study, including 66 tested in California [mean age = 36.6; Mean Mandarin proficiency = 4.48 (self-reported on a scale of 1 to 5), Mean English proficiency = 4.01] and 32 tested in Taiwan (mean age = 24.8; Mean Mandarin proficiency = 5.00, Mean English proficiency = 2.71).

### Materials and methods

We followed the three-dimensional pointing paradigm used in Fuhrman and Boroditsky (2010). The experimenter stood next to (and faced the same direction as) a participant, selected a spot in space directly in front of the participant (about a foot in front of the chest, with the palm facing up and the fingers brought together into a cone) and asked (for example) one of the test questions in Table 4. Participants pointed to locations in the space around them to locate these time points. Half of the participants were tested using front-back metaphors and half were tested using up-down metaphors. Participants in both conditions were asked to arrange weeks (up/down week and front/back week relative to this week) and months (up/down month and front/back month relative to this month), in that order. It is important to note that these are conventional metaphoric expressions in Mandarin, not novel constructions. Asking about the *up month* or *down month* in Mandarin, for example, is the analog of asking about the *last month* or *next month* in English. Further, there is no common non-spatial way to specify an earlier/later temporal relation in these cases, one would typically choose either a front-back or an up-down metaphor.

**Table 4:** **Example test questions using front-back and up-down metaphors in Mandarin**.

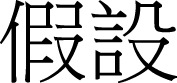	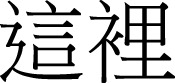	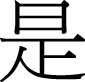	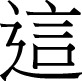	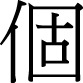	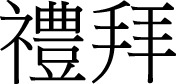		
jia-she	zhe-li	shi	zhe	ge	li-bai		
suppose	this here	is	this	classifier-ge	week		
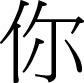	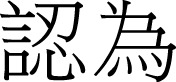	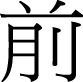	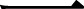	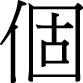	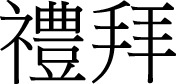	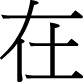	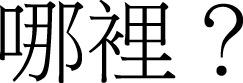
Ni	ren-wei	qian	yi	ge	li-bai	zai	na-li?
you	think	front	one	classifier-ge	week	locate	where?
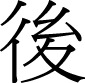	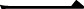	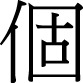	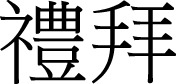	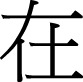	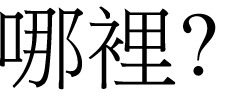		
Hou	yi	ge	li-bai	zai	nali?		
Back	one	classifier-ge	week	locate	where?		
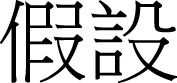	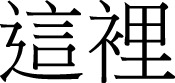	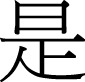	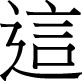	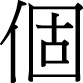	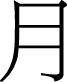		
jia-she	zheli	shi	zhe	ge	yue		
suppose	this here	is	this	classifier-ge	month		
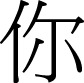	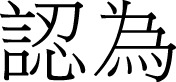	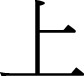	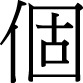	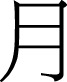	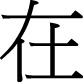	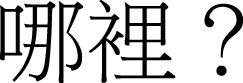	
ni	ren-wei	shang	ge	yue	zai	na-li?	
you	think	up	classifier-ge	month	locate	where?	
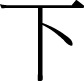	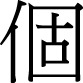	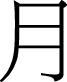	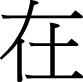	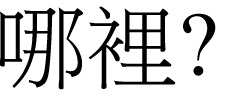			
xia	ge	yue	zai	na-li?			
down	classifier-ge	month	locate	where?			

All participants were tested in Mandarin by a native Mandarin-speaking experimenter. After the pointing task, participants filled out a language background questionnaire, listing the languages they speak, and how proficient they are in those languages on a scale from 1 to 5.

### Results

Data were coded using the same criteria used in Fuhrman and Boroditsky (2010, Exp 1), and were then grouped into three bins of interest: the front-back axis, the up-down axis, and the left-right axis.

**Figure 2:**
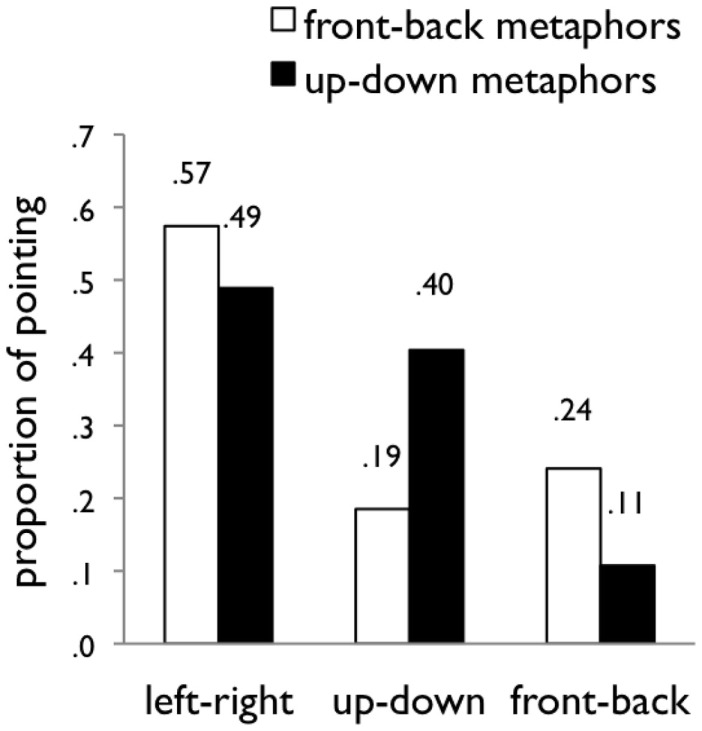
**Results of Study 2**. The *y*-axis indicates the proportion of arrangements that fell along the three axes (left-right, up-down, front-back), depending on whether the participant was cued with front-back or up-down metaphors.

**Figure 3:**
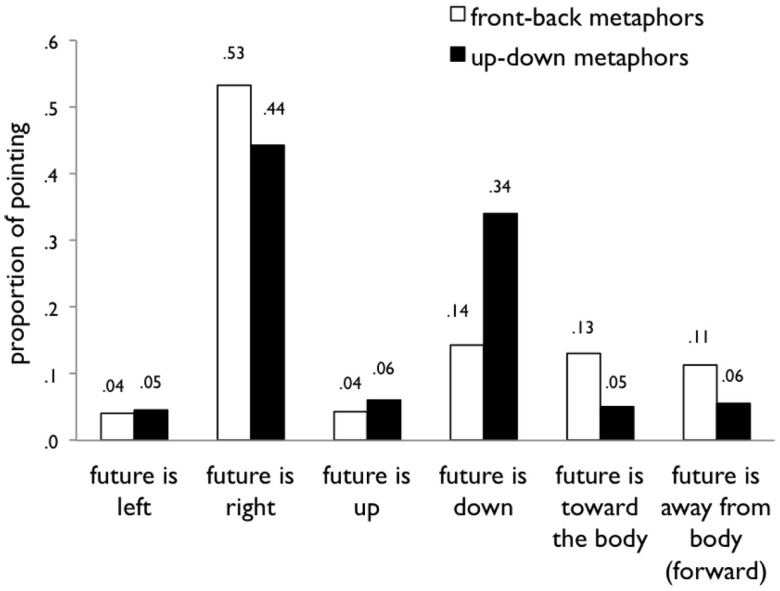
**Results of Study 2 broken down by direction**. The graph shows the proportion of arrangements in six directions (whether time was arranged with the future to the left, right, above, below, away from or toward the body with respect to the reference point) when participants were cued with front-back or up-down metaphors.

Results are summarized in Figure 2. Figure 3 shows the same data broken down by direction within each of the axes. To analyze the data, we fit linear regression models for each of the three axes (front-back, up-down, left-right) with the following three factors as predictors: (1) proficiency in Mandarin (one to five), (2) test location (California or Taiwan), and (3) metaphor (up-down or front-back). This set of three predictors captured a significant proportion of the variance in all three models. The regression results are reported in Table 5.

**Table 5:** **Results of linear regression analyses for each of the three axes (left-right, up-down, front-back) with the three factors as predictors: (1) Proficiency in Mandarin (2) Test location, and (3) Metaphor in Study 2**.

		Left-right	Up-down	Front-back
Mandarin fluency (1–5)	beta	−0.10	0.08	0.04
	t	−1.42	1.04	0.60
	p	0.16	0.30	0.55
Test location (California or Taiwan)	beta	−0.22	0.08	0.19
	t	*−2.96*	1.09	*2.51*
	p	0.00	0.28	0.01
Metaphor (up-down or front-back)	beta	0.10	−0.26	0.17
	t	1.45	*−3.66*	*2.39*
	p	0.15	0.00	0.02
ANOVA	F	*5.58*	*5.38*	*5.03*
	p	0.00	0.00	0.00
Adjusted R-squared		0.07	0.06	0.06

*Statistically significant results are indicated with asterisks*.

In sum, the metaphors mattered. Participants arranged time differently when prompted with front-back metaphors than when prompted with up-down metaphors in Mandarin. In particular, people were twice as likely to arrange time vertically when prompted with up-down metaphors (40%) as when prompted with front-back metaphors (19%), standardized β = −0.255, *p* < 0.0001. Further, people were more than twice as likely to arrange time sagitally (on the front-back axis) when prompted with front-back metaphors (24%) as when prompted with up-down metaphors (11%), β = 0.167, *p* < 0.05. Metaphors did not significantly affect arrangements along the left-right axis (β = 0.101, *p* = 0.15).

In addition, the test location mattered. Participants tested in California were more likely to use the left-right axis than those tested in Taiwan (61 and 36%, respectively; β = −0.218, *p* < 0.005) and less likely to use the front-back axis (12 and 29% respectively; β = 0.185, *p* < 0.05). Responses along the up-down axis did not differ significantly by test location (27 and 35% respectively; β = 0.081, *p* = 0.275). The difference between the two locations along the left-right axis is likely the result of differences in experience reading and writing text oriented from left to right (see Bergen and Lau, 2012).

The factor of Mandarin proficiency did not predict the participants’ preference for axis. This is likely because all of the participants included in this study were very proficient in Mandarin.

### Discussion

In this study we examined whether using different metaphors influences people’s representations of time in-the-moment. We found that indeed, Mandarin speakers were more likely to lay out time along the front-back axis when understanding front-back metaphors and more likely to lay out time vertically when understanding up-down metaphors^1^. With up-down metaphors, we saw a specific increase in how often Mandarin speakers placed earlier or past events above and later or future events below (see Figure 3). With front-back metaphors, we saw an increase in front-back arrangements in both directions: Mandarin speakers were equally likely to place the past further in front as they were to place the future further in front.

The pattern of results we observe along the front-back axis replicates previous such patterns observed with Mandarin speakers on this task. For example, Fuhrman et al. (2011)compared English and Mandarin speakers on the same time-pointing task, but using non-spatial language (terms like yesterday, today, tomorrow) as prompts instead of explicit spatial metaphors. English speakers mostly arranged time on the left-right axis (93.5%) with up-down and front-back arrangements being much less frequent (2.5 and 3.9% respectively). Mandarin speakers tested in Mandarin were about equally likely to arrange time on the left-right axis (46.8%) as on the up-down axis (43.6%), with front-back arrangements making up the remaining 9.6%. While front-back arrangements were infrequent in both language groups, there was a significant difference in how participants laid out time on this front-back axis across the two language groups. Of the front-back arrangements, Mandarin speakers arranged time with the past further in front 41% of the time, whereas this pattern was negligible in English speakers.

What might be responsible for this flexibility in temporal arrangements along the front-back among the Mandarin speakers? One possibility suggested in the literature is that while in English the observer is always facing the future, in Mandarin the observer may sometimes be facing the past. For example, Lai (2002) and Ahrens and Huang (2002) suggest that in the time-moving scenario in Mandarin, the observer is facing the past with time washing over them from behind (in the ego-moving scenario, the observer is still facing the future as in English) (see also Núñez and Sweetser, 2006, for their case in the Aymara language).

These analyses are based on the interpretation of linguistic examples, however alternative interpretations of the examples are also possible. Consider Example 7 in Table 1. The “front year” in Mandarin is “2 years ago.” Some researchers have suggested this as linguistic evidence that the observer is facing the past, such that past events are in front of the observer and future events are behind (Ahrens and Huang, 2002; Lai, 2002; Zhang and Rong, 2007). An alternative analysis is that *qian* (front) and *hou* (back) function as adjectives modifying the stream of events in a timeline, implying that the temporal events themselves have a front and back. Since temporal events move from the future to the past (in the time-moving framework), the front of the timeline faces the past and the back side faces the future (Yu, 1998; Dong, 2004).

Mandarin speakers’ patterns of responses on the front-back axis in our pointing task suggest that Mandarin speakers do spontaneously conceptualize time both with the past further in front of the body and with the future further in front of the body. However, since most participants created their full temporal arrangements in the space in front of their bodies (placing events forward or back with respect to the reference point, but rarely pointing behind the body), results from a different task would be necessary to see if the future is indeed sometimes seen as behind one’s back.

## General Discussion

In this paper we have examined both chronic and in-the-moment consequences of metaphor use in constructing people’s representations of time.

In Study 1 we compared temporal reasoning in three groups with different histories of linguistic experience with time metaphors: English monolinguals, Mandarin monolinguals, and ME bilinguals. We find that English and Mandarin monolinguals indeed tend to take different perspectives on time, with Mandarin speakers more likely to take the time-moving perspective, consistent with the linguistic analyses of metaphor use in the two languages. Further, we find that ME bilinguals differ from both groups of monolinguals. When understanding time metaphors in English, ME bilinguals are more likely to adopt the time-moving perspective than are English monolinguals. When understanding time metaphors in Mandarin, ME bilinguals are less likely to adopt the time-moving perspective than are Mandarin monolinguals. That is, there are both effects of L1 on meaning-making in L2, and the reverse, effects of L2 on meaning-making in L1.

In Study 2, we test whether using different spatio-temporal metaphors can in-the-moment give rise to different representations of time. We find that Mandarin speakers are more likely to construct front-back representations of time when understanding front-back metaphors, and more likely to construct up-down representations of time when understanding up-down metaphors.

Taken together, these findings demonstrate that the metaphors we use to talk about time have both immediate and long-term consequences for how we conceptualize and reason about this fundamental domain of experience. How people conceptualize time appears to depend on how the languages they speak tend to talk about time, and also on the particular metaphors being used to talk about time in-the-moment.

## Conflict of Interest Statement

The authors declare that the research was conducted in the absence of any commercial or financial relationships that could be construed as a potential conflict of interest.

## Footnotes

^1^In this study, we used contrasting conventional spatial metaphors in Mandarin as part of the instructions and observed that these different metaphors generated different behavior. One interpretation of these results is that processing and understanding these conventional metaphors naturally lead participants to generate different spatial representations of time. Another possibility is that participants perceived the metaphors as explicit instructions about how to spatialize time for the purpose of the experiment, and so responded accordingly. There are a number of reasons that suggest this was not the case. First, the metaphors used in the study are canonical expressions in Mandarin, and no non-spatial equivalents exist. Because the design is between-subjects, the participants had no reason to suspect these conventional natural language metaphors as an experimental manipulation. Further, the large number of responses on the left-right axis suggests that participants did not take the metaphors used in the study to be explicit instructions. The metaphors used only front/back or up/down language and yet we observed a large proportion of responses on the left/right axis. Nonetheless this alternative take remains an important possibility. Studies that rely on less explicit measures of behavior will be necessary to further tease apart these alternative explanations.
